# Assessment of Hepatocellular Carcinoma Metastasis Glycobiomarkers Using Advanced Quantitative N-glycoproteome Analysis

**DOI:** 10.3389/fphys.2017.00472

**Published:** 2017-07-07

**Authors:** Tianhua Liu, Shuxin Shang, Wei Li, Xue Qin, Lu Sun, Shu Zhang, Yinkun Liu

**Affiliations:** ^1^Key Laboratory of Carcinogenesis and Cancer Invasion, Ministry of Education, Liver Cancer Institute, Zhongshan Hospital, Fudan UniversityShanghai, China; ^2^Institutes of Biomedical Sciences, Fudan UniversityShanghai, China; ^3^Department of Clinical Laboratory, First Affiliated Hospital of Guangxi Medical UniversityNanning, China

**Keywords:** hepatocellular carcinoma, metastasis, N-glycosite occupancy, lectin, tandem ^18^O stable isotope labeling

## Abstract

Hepatocelluar carcinoma (HCC) is one of the most common malignant tumors with high incidence of metastasis. Glycosylation is involved in fundamental molecular and cell biology process occurring in cancer including metastasis formation. In this study, lectin microarray, lectin blotting, lectin affinity chromatography and tandem ^18^O stable isotope labeling coupled with liquid chromatography-mass spectrometer (LC-MS) analysis were applied to quantify the changes in N-glycosite occupancy for HCC metastasis serum. Firstly, lectin microarray was used to screen glycoforms and Phaseolus vulgaris Leucoagglutinin (PHA-L) reactive structure (β1,6-GlcNAc branched N-glycan) was found to be increased significantly in HCC patients with metastasis compared with those with non-metastasis. Then, PHA-L affinity glycoproteins were enriched followed by N-glycosite occupancy measurement with strategy of tandem ^18^O stable isotope labeling. 11 glycoproteins with significantly changed N-glycosite occupancy were identified, they were associated with cell migration, invasion and adhesion through p38 mitogen-activated protein kinase signaling pathway and nuclear factor kappa B signaling pathway. Quantification of N-glycosite occupancy for PHA-L reactive glycoproteins could help to discover important glycoproteins of potential clinically significance in terms of HCC etiology. Also, understanding of N-glycosite occupancy alterations will aid the characterization of molecular mechanism of HCC metastasis as well as establishment of novel glycobiomarkers.

## Introduction

Hepatocellular carcinoma (HCC) as the major primary liver cancer is the third leading cause of cancer-related death and account for 70–85% of the liver cancers worldwide (Jemal et al., 2011). Despite the medical techniques have experienced significant advances, the clinical prognosis still remains extremely poor and the 5-year survival rate in HCC patients after surgical resection is only 20–30% (Yamamoto et al., 2001; Pang et al., 2008; Yang et al., 2013). High incidences of recurrence and metastasis rate contribute to the long-term prognosis unsatisfactory (Tung-Ping Poon et al., 2000; Jia et al., 2011). HCC metastasis was due to the comprehensive effect of manifold causes and always began by HCC cells breaking through the walls of nearby lymph or blood vessels. It is very important to identify the changed biomolecular associated with HCC metastasis so that it could predict the risk of metastasis and the prognostic value, which may contribute to better treatments for the patients.

Glycosylation is one of the most prominent forms of posttranslational protein modification and more than 50% of human protein are presumed to have undergone glycosylation (Apweiler et al., 1999). Glycosylation plays a major role in regulating critical cellular functions and assembly of complex multicellular organs and organisms. It is involved in cell-cell and receptor-ligand interactions, signal transduction, and endocytosis (Varki and Lowe, 2009; Karve and Cheema, 2011; Rakus and Mahal, 2011). Abnormal glycosylation is associated with malignant transformation (Kannagi et al., 2004; Mi et al., 2012). In recent years, a handful of glycoproteins as cancer biomarkers have completed the program from discovery to verification and validation (Kim and Misek, 2011; Kuzmanov et al., 2013; Shah et al., 2015). One typical example is the Lens Culinaris Agglutinin-reactive fraction of alpha-fetoprotein (AFP-L3). The tumor marker AFP is widely used for HCC's surveillance (Blomme et al., 2009; Xia et al., 2012), while on account of AFP-negative HCC is frequently observed, AFP-L3 has been a preferred HCC biomarker in early diagnosis of HCC and in predicting prognosis after treatment (Sato et al., 1993; Okuda et al., 1999; Kumada et al., 2014). It was reported that complement C3, ceruloplasmin, histidine-rich glycoprotein, CD14, hepatocyte growth factor (HGF) (Liu, Y. et al., 2010), hemopexin, fetuin-A (Comunale et al., 2009) and haptoglobin (Zhang et al., 2016) could be potential glycobiomarkers for distinguishing HCC. However, some glycobiomarkers are not unique for HCC progression, which could be observed in most gastrointestinal (GI) cancers and may provide clinical assistant diagnosis for HCC (Dempsey and Rudd, 2012; Ren et al., 2016).

Glycoproteins have been found to play important roles in invasion and metastasis of tumors. Accurate characterization of glycoproteins with multiple glycosylation sites and assessment of the glycan macroheterogeneity (glycosite occupancy) and microheterogeneity (glycan structure) are urgently needed for understanding the functions of glycans in HCC. Especially, N-glycosite occupancy is associated with the enzymatic activity and the physical stability of glycoproteins (Baboval et al., 2000; Alsenaidy et al., 2014), which might contribute to the metastasis of HCC. An endoplasmic reticulum-retained green fluorescent protein (GFP) biomarker was reported, whose fluorescence was lost when it was N-glycosylated. This marker was a highly sensitive indicator of N-glycosite occupancy of multiple cell lines. But it could not be used to measure glycosite occupancy of other target glycoproteins (Losfeld et al., 2012). Xu et al. (2015) developed SWATH-MS-based methods were developed for automated measurement of glycosite occupancy in N-glycoproteins from the yeast cell wall and from human whole saliva. Sumer-Bayraktar et al. (2012) performed a MS-driven glycoproteomics and glycomics combined with exoglycosidase treatment to determine glycosite occupancies of serum-derived Hshbg. A universal workflow for site-specific N- and O-glycopeptide analysis of pronase treated glycoproteins was described and glycosite occupancy of IgG3 was reported (Stavenhagen et al., 2015).

A novel strategy using tandem ^18^O stable isotope labeling (TOSIL) could quantify N-glycosite occupancy by measuring the intensity ratios of ^18^O/^16^O for glycosylated (6 Da) and for non-glycosylated (4 Da) peptides (Liu, Z. et al., 2010). This method could quantify the changes of N-glycosite occupancy in complex protein mixtures and produce a 6 Da difference among differently labeled glycopeptides which was easily observed. In this study, lectin microarray was used to screen metastasis-related glycoforms which were validated by lectin blotting analysis. PHA-L reactive structure (β1,6-GlcNAc branched N-glycan) was found to be increased significantly in HCC patients with metastasis compared with those with non-metastasis. Then, PHA-L affinity glycoproteins were enriched and 11 glycoproteins with changed N-glycosite occupancy were identified using TOSIL strategy coupled with LC-MS analysis. What was more, p38 mitogen-activated protein kinase signaling pathway (p38 MAPK) and nuclear factor kappa B signaling pathway (NF-κB) were found to be significant nodes in IPA network, indicating that these glycoproteins played important roles in biological processes of HCC metastasis.

## Materials and methods

### Clincial specimens

Serum samples from 80 HCC patients were collected at First Affiliated Hospital of Dalian Medical University and stored at −80°C. The clinicopathological data of the patients were provided in Table 1. Pooling sera of 10 HCC patients with extrahepatic metastasis (metastasis) and 10 HCC patients with non-metastasis were used for analyses. Four biological repeats were measured independently to guarantee the reproducibility of experiment. Written informed consent was obtained from each patient. All subjects gave written informed consent in accordance with the Declaration of Helsinki. The protocol was approved by the Ethics Committee of First Affiliated Hospital of Dalian Medical University. All methods in this study were performed in accordance with the human experimentation guideline of the People's Republic of China.

**Table 1:** General information and clinical characteristics of HCC patients for screening.

	**Non-metastatic *n* = 40**	**Metastatic *n* = 40**	***p***
Age (years)	56 ± 10	57 ± 12	0.855^a^
Gender (male/female)	37 (92.5%)/3 (7.5%)	38 (95%)/2 (5%)	0.644^b^
AFP (IU/ml)	353.2 ± 332.7	437.3 ± 326.6	0.578^a^
ALT (IU/L)	52.7 ± 42.1	59.3 ± 43.2	0.708^a^
AST (IU/L)	71.3 ± 44.1	81.5 ± 65.7	0.703^a^
HbsAg (yes/no)	36 (90%)/4 (10%)	39 (97.5%)/1 (2.5%)	0.166^b^
PT (s)	13.2 ± 1.8	14.7 ± 2.5	0.121^a^

a *Student's T-test*.

b Chi-square test

*The values supplied were mean ± standard deviation. AFP, Alpha fetoprotein; ALT, Alanine aminotransferase; AST, Aspartate transaminase; HbsAg, Hepatitis B surface antigen; PT, Prothrombin time*.

Each pooling sera was mixed by equivalent volume of individual sera (6 μL), and the total volume of pooling sera was 60 μL. Albumin and IgG were depleted by ProteoExtract® Albumin/IgG removal kit (Calbiochem, Billerica, MA, USA) from pooling sera according to the manufacturer's description.

### Lectin microarray analysis

One microgram proteins were biotinylated by Lightning-Link Biotin Labeling Kit (Innova Biosciences, Cambridge, UK). A lectin microarray was produced using 50 lectins (Vector Laboratories, Burlingame, CA, USA; Sigma-Aldrich, Castle Hill, NSW, Australia). The name and the binding specificity of 50 lectins were provided in Table S1. The workflow for lectin microarray was described in Figure S1: after blocking the non-specific binding sites with 2% bovine serum albumin (BSA)-phosphate buffer saline (PBS), the lectin microarray was incubated with equal biotinylated proteins (non-metastatic or metastatic) and Cy5 labeled streptavidin (Life technologies, Waltham, MA, USA) in turn. LuxScan 10K/A scanner system (CapitalBio, Beijing, China) was used to scan and data were analyzed as described previously (Xin et al., 2014).

### Lectin blotting analysis

Twenty microgram proteins for identification were separated by SDS-PAGE and transferred onto PVDF membranes (Millipore, Billerica, MA, USA). After blocking, the membranes were incubated with biotinylated Datura Stramonium Agglutinin (DSA), Maackia Amurensis Lectin-I (MAL-I), PHA-L and Wheat Germ Agglutinin (WGA) (Vector Laboratories, Burlingame, CA, USA), respectively. The membranes were washed with 0.1% TBS-Tween20 (TBST, 50 mM Tris, 150 mM NaCl, 0.1% Tween 20, pH 7.6) and then incubated with Streptavidin Horseradish Peroxidase (HRP) Conjugate (Invitrogen, Waltham, MA, USA). Amersham ECL prime western blotting detection reagents (GE Healthcare, Piscataway, NJ, USA) were used to detect the bands on the membranes.

### Lectin affinity chromatography

PHA-L agarose was washed and resuspended with the lectin-binding solution (10 mM Tris-HCl, pH7.5, 0.15 M NaCl, 1 mM CaCl_2_, 1 mM MgCl_2_). Then, 3 mg proteins from different assemblages (non-metastatic or metastatic) were added into PHA-L agarose and incubated at 4°C overnight with a round shaker. Lectin-binding solution was used to wash the agarose and the bound fraction was eluted by 200 mM N-acetyl-D-(+)-glucosamine. The eluted fraction was separated by SDS-PAGE and stained by PhastaGel™ Blue R. The gels containing all bands were cut and processed for in-gel digestion.

### In-gel digestion and strategy of tandem ^18^O stable isotope labeling

The destained gel pieces were reduced and alkylated with Tris-(2-carboxy-ethyl)-phosphine hydrochloride (TCEP, Sigma, Castle Hill, NSW, Australia) and iodoacetamide (IAA, Sigma, Castle Hill, NSW, Australia), respectively. Subsequently, the gel pieces were re-dehydrated with 100% ACN, and then digested in trypsin solution (5 ng/μL) at 37°C overnight. TOSIL strategy was performed as described previously (Liu, Z. et al., 2010), and the workflow was shown in Figure S2. HCC patients with metastasis were treated in ${\text{H}}_{2}^{18}$O and those with non-metastasis treated in ${\text{H}}_{2}^{16}$O.

### LC-MS analysis

The experiments were performed on a Nano Aquity UPLC system (Waters Corporation, Milford, MA, USA) connected to a quadrupole-Orbitrap mass spectrometer (Q-Exactive) (Thermo Fisher Scientific, Bremen, Germany) equipped with an online nano-electrospray ion source. The Q-Exactive mass spectrometer was operated in the data-dependent mode to switch automatically between MS and MS/MS acquisition. Survey full-scan MS spectra (m/z 350–1,800) were acquired with a mass resolution of 70 K, followed by 10 sequential high energy collisional dissociation (HCD) MS/MS scans with a resolution of 17.5 K. In all cases, one microscan was recorded using dynamic exclusion of 30 s.

### Datebase searching and quantification

The deglycosylated glycopeptides and non-glycosylated peptides were searched against SWISS-PROT human database using the MaxQuant 1.5.3.30, a quantitative proteomics software package. The parameters for searching were set: enzyme, partial trypsin; missed cleavages allowed, two; fixed modification, carboxyamidomethyla-tion (Cys); variable modifications, deamidation (Asn), deamidation plus ^18^O (Asn), ^18^O C-term and oxidation (Met); peptide tolerance, 10 ppm; MS/MS tolerance, 0.05 Da. The relative quantities of N-glycosylated and its parent protein levels were obtained simultaneous by measuring the intensity ratios of ^18^O/^16^O for glycosylated (6 Da) and for non-glycosylated (4 Da) peptides from the same proteins respectively. A comparison of these two ratios can be utilized to evaluate the change of N-glycosite occupancy between HCC patients with metastasis (^18^O labeling) and those with non-metastasis (^16^O labeling) by the Equation as follows:

$$\begin{array}{l} \text{Change of N-glycosite occupancy }=\;\frac{\text{Intensity of}{\;}^{18}\text{O deglycosylated glycopeptide}/\text{Average intensity of}{\;}^{18}\text{O non-glycosylated peptides}}{\text{Intensity of}{\;}^{16}\text{ O deglycosylated glycopeptide}/\text{Average intensity of}{\;}^{16}\text{O non-glycosylated peptides}}\\ \;=\;\frac{\text{Intensity of}{\;}^{18}\text{O deglycosylated glycopeptide}}{\text{Intensity of}{\;}^{16}\text{O deglycosylated glycopeptide}}\\ \;\times \;\frac{\text{Average intensity of}{\;}^{18}\text{O non-glycosylated peptides}}{\text{Average intensity of}{\;}^{16}\text{O non-glycosylated peptides}}\\ \;=\;\frac{{\;}^{18}\text{O}{/}^{16}\text{O ratio for deglycosylated glycopeptide}}{\;{\;}^{18}\text{O}{/}^{16}\text{O ratio for protein}} \end{array}$$

Fold changes >1.5 or <0.667 were considered to be significant. Fold changes between 1.2 and 1.5 (1.2–1.5) or between 0.667 and 0.833 (0.667–0.833) were considered as minor.

### Functional annotation and patterns analysis

Functional categories of 11 N-glycoproteins with changed N-glycosite occupancy were analysis using OmicsBean (http://www.omicsbean.com). Ingenuity Pathway Analysis (IPA) analysis (QIAGEN, Redwood City, CA, USA) was used to investigate biological interactions. Motif extractor (Motif-X, http://motif-x.med.harvard.edu) was used to identify overrepresented glycosylation motifs. Proteins annotated in International Protein Index (IPI) human proteome database were used as background and the significance value was set as 0.000001 (Schwartz and Gygi, 2005; Chou and Schwartz, 2011; Wang et al., 2014; Zhang et al., 2015).

## Results

### Altered glycoforms of HCC metastasis sera

**Figure 1:**
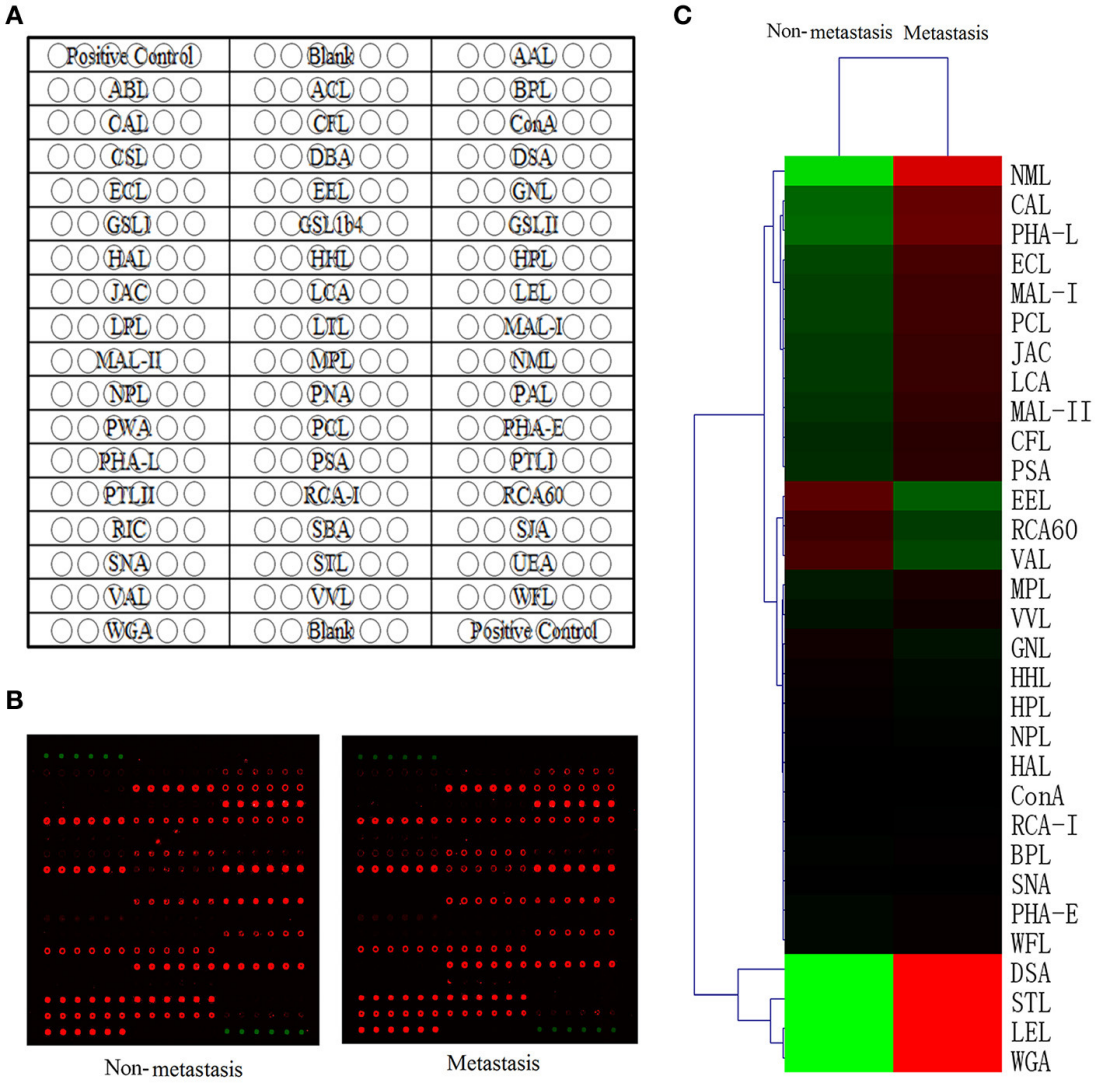
Lectin microarray analysis of glycoforms. **(A)** The lectin microarray contains 50 lectin spots with different binding specificities. **(B)** Scan image of the lectin microarray incubated with biotinylated proteins and Cy5 labeled streptavidin. Typical glycan profiles of HCC with metastasis were shown on the right and HCC with non-metastasis on the left. **(C)** Hierarchical clustering of positive lectin binding spots (S/B≥2). Each row represented a single lectin, S/B values were shown by the color scale: red represents a lectin with high S/B value while green represents a lectin with low S/B value.

A high-throughput lectin microarray (Figure 1A) which included 50 lectins, 2 positive controls and 2 blank controls in each block was applied to detect different glycoforms between HCC patients with metastasis and HCC patients with non-metastasis. The positive controls were albumin coupling with Cy3 which showed by Cy3 scanning, and the blanks in each block were the negative controls (Figure 1B). The Spot Intensity Median (S) and the Background Intensity Median (B) were extracted and S/B was calculated. Using S/B ≥2 as cutoff, 31 lectins were defined as positive lectin binding spots. Hierarchical clustering of them was mapped by The MeV 4.8.1. (Figure 1C).

Among these 31 lectins, 14 lectins had statistical significance (*p* < 0.05) and we divided protein-lectin binding intensities of them into 3 grades: weak binding (5>S/B ≥2), medium binding (15>S/B≥5) and strong binding (S/B≥15). In non-metastatic HCC samples, Caragana Arborescens Lectin (CAL), Euonymus Europaeus Lectin (EEL), MAL-I, Maackia Amurensis Lectin-II (MAL-II) were weak binding; Erythrina Cristagalli Lectin (ECL), Galanthus Nivalis Lectin (GNL) and Lens Culinaris Agglutinin (LCA) were medium binding; DSA, Lycopersicon Esculentum Lectin (LEL), Naja Mossambica Lectin (NML), Phaseolus Coccineus Lectin (PCL), PHA-L, Solanum Tuberosum Lectin (STL), and WGA were strong binding. While, in metastatic samples, EEL, MAL-I, MAL-II were weak binding; CAL, ECL, GNL, and LCA were medium binding; DSA, LEL, NML, PCL, PHA-L, STL, and WGA were strong binding.

**Figure 2:**
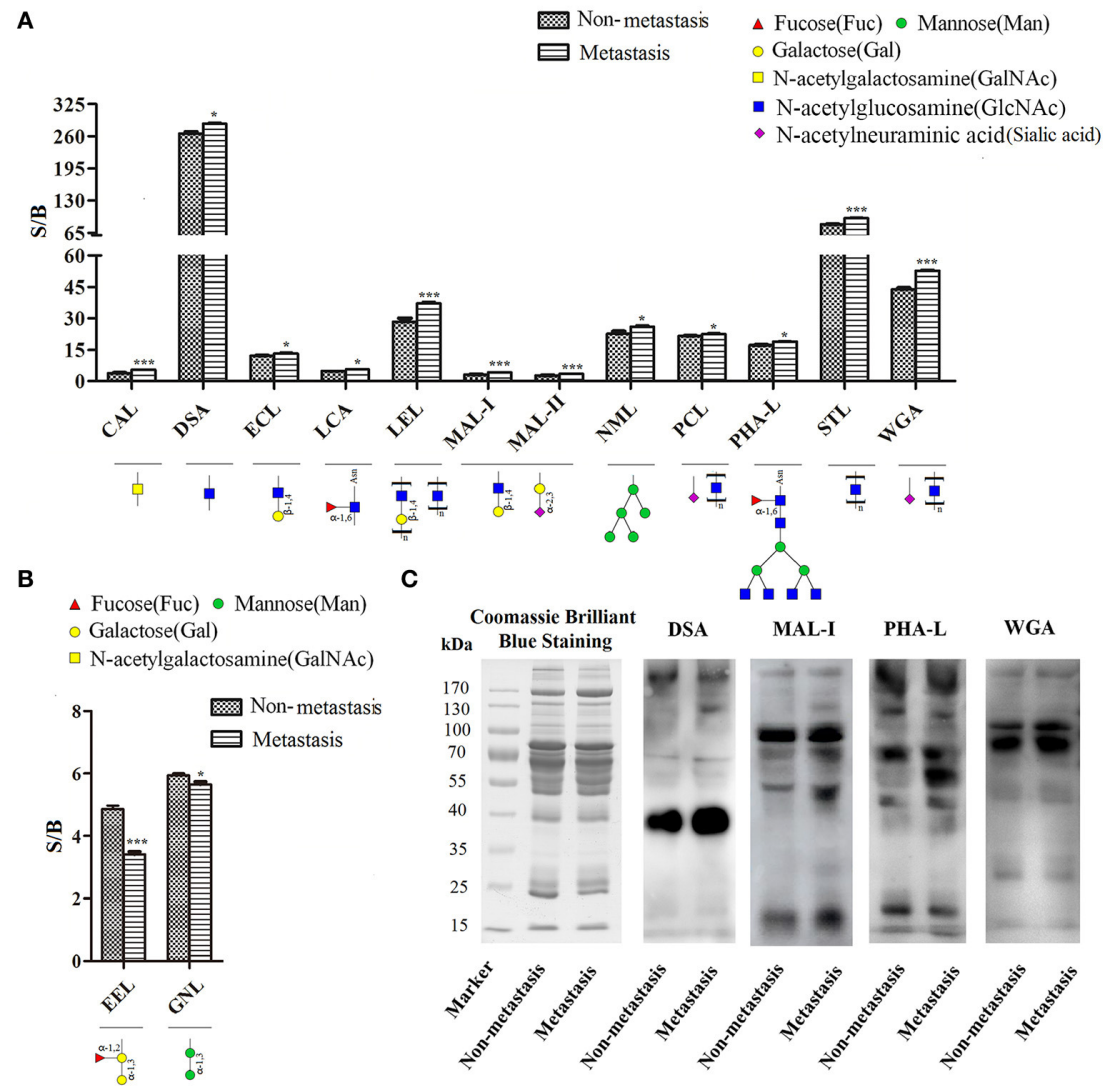
Screening and identification of changes in glycoforms of serum glycoproteins. **(A)** Specific binding abilities and quantitative results of lectins with significant up-regulated binding capacity in metastatic samples. **(B)** Specific binding abilities and quantitative results of lectins with significant down-regulated binding capacity in metastatic samples. ^*^p < 0.05, ^***^p < 0.001. **(C)** Lectin blotting by biotinylated lectins: DSA, MAL-I, PHA-L, and WGA. Coomassie brilliant blue staining by PhastaGel™ Blue R showed similar global abundance of serum proteins in HCC patients with metastasis and those with non-metastasis. DSA, MAL-I, PHA-L, and WGA binding glycoforms were increased in HCC patients with metastasis compared with those with non-metastasis, which were consistent with the results of lectin microarray.

Quantitative results of S/B and specific binding abilities of the 14 lectins were shown in Figures 2A,B, 12 lectins: GalNAc binder CAL, GlcNAc binder DSA and STL, β-1,4Gal binder ECL, Fucα-1,6GlcNAc binder LCA, Poly-LacNAc or (GlcNAc)n binder LEL, α-2,3Sia or β-1,4Gal binder MAL-I and MAL-II, exopolysaccharide binder NML, Sia binder PCL, β1,6-GlcNAc branched N-glycan binder PHA-L and (GlcNAc)n or multivalent Sia binder WGA showed increasing trend in metastatic HCC samples compared to non-metastatic HCC samples; However, α-1,3Gal binder EEL and α-1,3mannose binder GNL were lectins showed decreasing trend. Among them, the *p*-values of lectins CAL, LEL, MAL-I, MAL-II, STL, WGA, and EEL were less than 0.001, while, the *p*-values of lectins DSA, ECL, LCA, NML, PCL, PHA-L, and GNL were less than 0.05. It suggested that structures such as GalNAc, GlcNAc, β-1,4Gal, Fucα-1,6(GlcNAc)n, Sia and β1,6-GlcNAc branched N-glycan were increased significantly in HCC patients with metastasis; while, α-1,3Gal and α-1,3mannose were decreased significantly.

### Confirmation of the changed glycoforms by lectin blotting

Lectin blotting was performed to validate changed glycoforms using biotinylated lectin DSA, MAL-I, PHA-L, and WGA. Coomassie brilliant blue staining showed similar global abundance of serum proteins in HCC patients with metastasis and those with non-metastasis. GlcNAc (which binds to DSA), α-2,3Sia or β-1,4Gal (which binds to MAL-I), β1,6-GlcNAc branched N-glycan (which binds to PHA-L) and (GlcNAc)n or multivalent Sia (which binds to WGA) were increased in HCC patients with metastasis compared with those with non-metastasis, which were consistent with the results of lectin microarray (Figure 2C).

Among them, β1,6-GlcNAc branched N-glycan was significantly changed. This structure was catalyzed by UDP-N-acetylglucosamine: α-6-D-manno-side β1–6-N-acetylglucosaminyltransferase (EC2.4.1.155) which was known as GnT-V. Expression levels of β1,6-GlcNAc branched N-glycan and GnT-V were associated with metastasis in human digestive cancers such as colorectal carcinoma and gastric cancer (Seelentag et al., 1998; Kim et al., 2008; Huang et al., 2013; Huang, B. et al., 2014). In our previous studies, we have found this glycoform was increased in epithelial mesenchymal transition (EMT) process of Huh7 HCC cell and it might be a metastasis-promoting glycoform in HCC (Li, S. et al., 2013).

### Quantification of N-glycosite occupancy for PHA-L reactive glycoproteins

**Figure 3:**
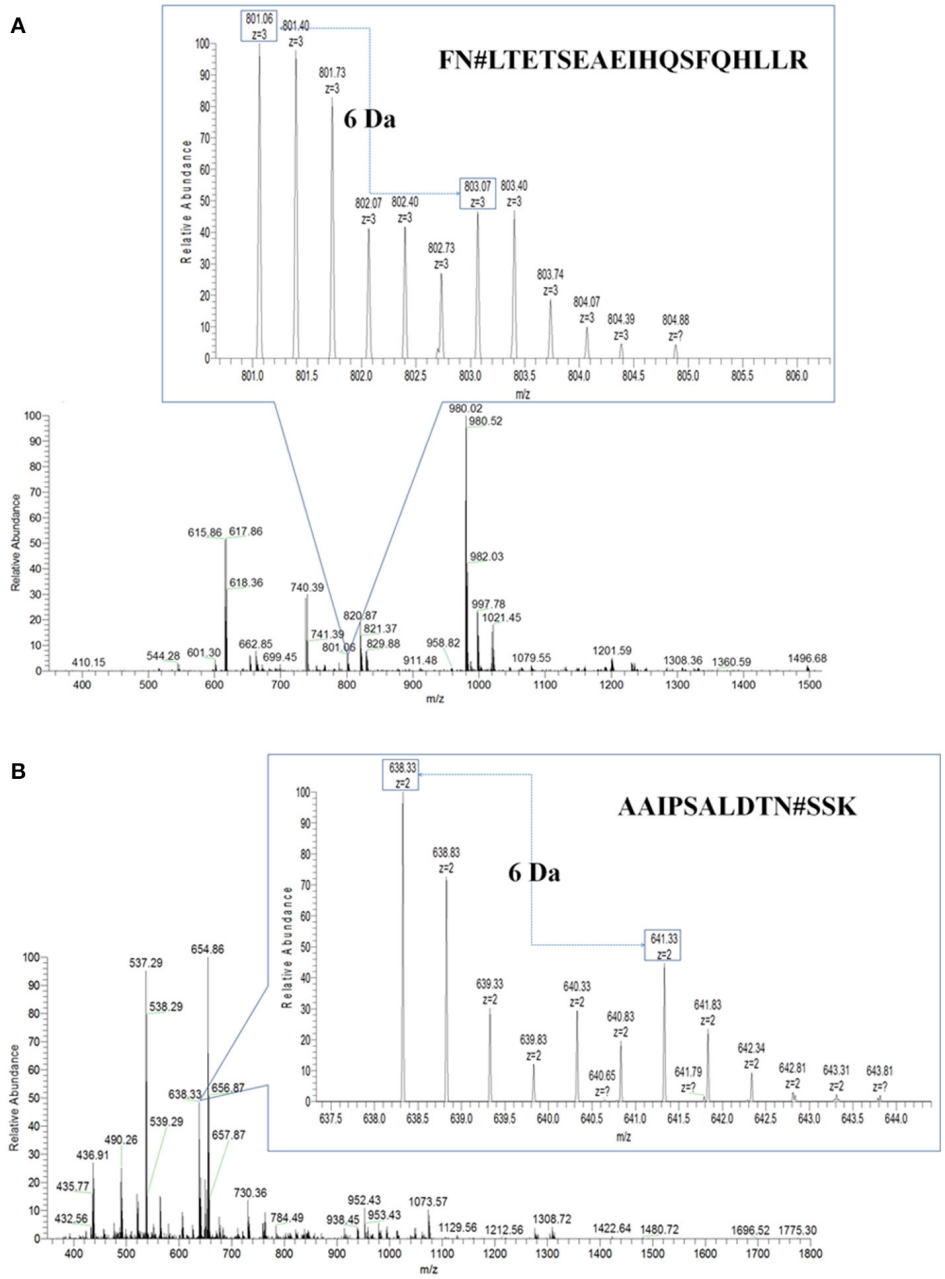
Deglycosylated glycopeptides were examined by the TOSIL strategy with LC-MS. **(A)** Quantitative analysis of deglycosylated glycopeptides FN#LTETSEAEIHQSFQHLLR from alpha-1-antichymotrypsin by LC-MS. **(B)** Quantitative analysis of deglycosylated glycopeptides AAIPSALDTN#SSK from fibulin-1 by LC-MS. A unique mass shift of 6 Da was shown for N-glycosylated peptide with single glycosylation site.

**Figure 4:**
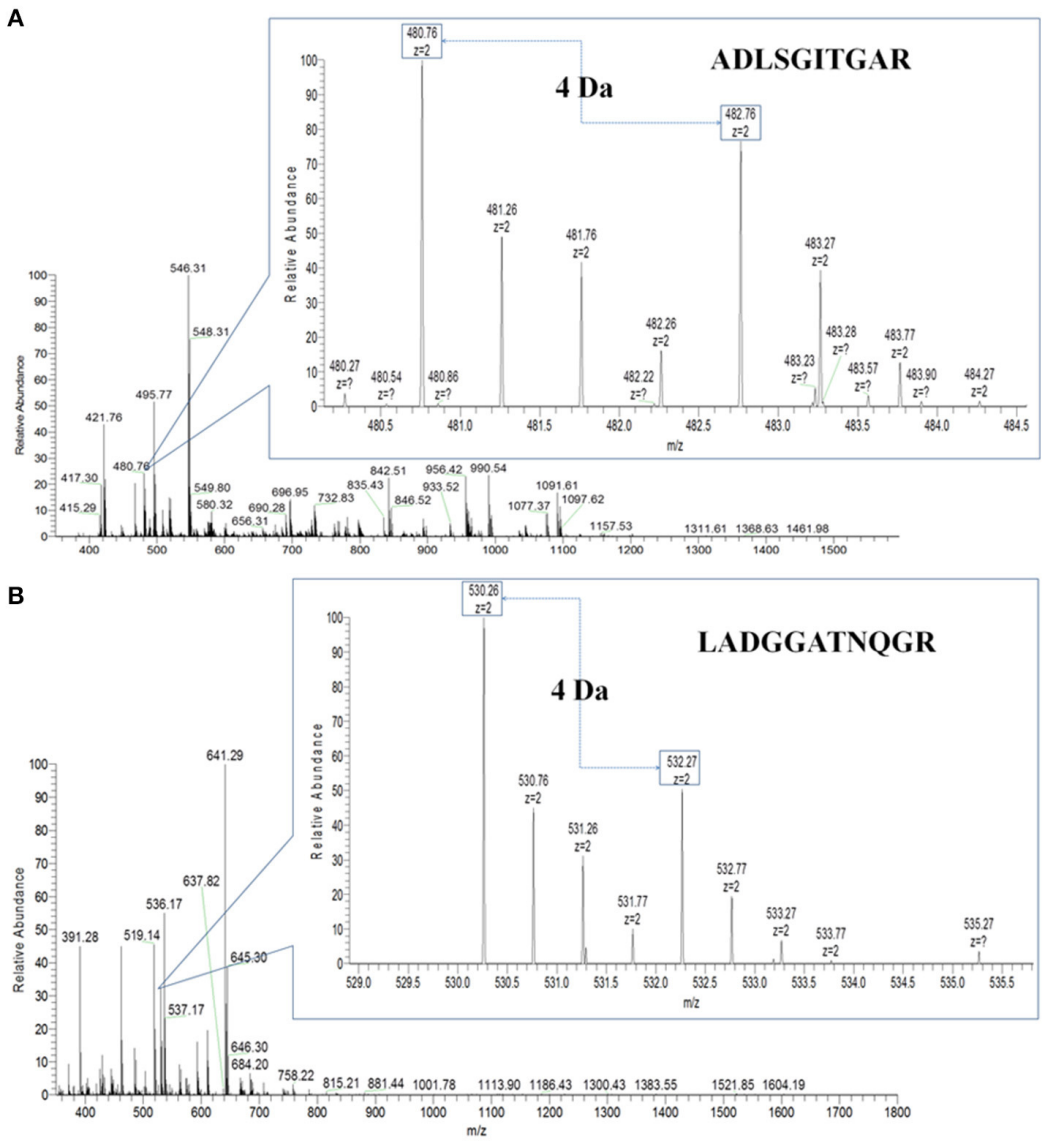
Non-glycosylated peptides were examined by the TOSIL strategy with LC-MS. **(A)** Quantitative analysis of non-glycosylated peptide ADLSGITGAR from alpha-1-antichymotrypsin by LC-MS. **(B)** Quantitative analysis of non-glycosylated peptide LADGGATNQGR from fibulin-1 by LC-MS. A mass difference of 4 Da was shown for nonglycosite peptide.

Then, PHA-L affinity chromatography was chosen to enrich serum N-glycoproteins and a total of deglycosylated glycopeptides from 14 glycoproteins were quantified in HCC patients with metastasis compared with those with non-metastasis (Table 2). The cutoff of fold change was determined by experiments: the same sera sample was divided into two equal parts for ^16^O/^18^O labeling, which indicated expected ratio of 1:1 (fold change = 1). The average (five replicates) measured ratios of N-glycosite occupancy was 1:1.19 (fold change = 1.19), which indicated the cutoff of fold change was 1.19. Considering complexity of sera, the cutoff was set as 1.5 (data not shown). Among these deglycosylated glycopeptides, there were 6 deglycosylated glycopeptides displayed significant changes in N-glycosite occupancy (fold changes>1.5 or <0.667, highlighted in bold) and 7 deglycosylated glycopeptides with minor changes (fold changes 1.2–1.5 or 0.667–0.833, highlighted in italics). Figure 3 showed representative MS spectra of deglycosylated glycopeptides FN#LTETSEAEIHQSFQHLLR from alpha-1-antichymotrypsin and AAIPSALDTN#SSK from fibulin-1. MS spectra of non-glycosylated peptides ADLSGITGAR from alpha-1-antichymotrypsin and LADGGATNQGR from fibulin-1 were shown in Figures 4A,B, respectively. Characteristic 6 or 4 Da shift in mass could be observed via TOSIL strategy.

**Table 2:** Changes of N-glycosite occupancy in HCC patients with metastasis compared with those with non-metastasis.

**UniProt number**	**Protein name**	**Peptide sequence^a^**	**^18^O/^16^O ratio for deglycosylated glycopeptide**	**Average ^18^O/^16^O ratio for protein**	**Change in N-glycosite occupancy (Metastasis/Non-metastasis)^b^**
P00450	Ceruloplasmin	EHEGAIYPDN#TTDFQR	0.483	0.573	0.843
		*EN#LTAPGSDSAVFFEQGTTR*	*0.767*	*0.573*	*1.340*
P00738	Haptoglobin	**VVLHPN#YSQVDIGLIK**	**1.300**	**0.852**	**1.525**
P01008	Antithrombin-III	**SLTFN#ETYQDISELVYGAK**	**0.446**	**0.915**	**0.487**
P01009	Alpha-1-antitrypsin	**YLGN#ATAIFFLPDEGK**	**0.438**	**0.736**	**0.595**
P01011	Alpha-1-antichymotrypsin	*FN#LTETSEAEIHQSFQHLLR*	*0.658*	*0.913*	*0.721*
P02790	Hemopexin	SWPAVGN#CSSALR	0.577	0.498	1.159
P07996	Thrombospondin-1	VVN#STTGPGEHLR	1.587	1.421	1.117
P08603	Complement factor H	**ISEEN#ETTCYMGK**	**0.394**	**0.800**	**0.492**
P0C0L5	Complement C4-B	**GLN#VTLSSTGR**	**0.254**	**0.525**	**0.484**
P10909	Clusterin	**KKEDALN#ETR**	**0.456**	**0.957**	**0.476**
		*HN#STGCLR*	*0.722*	*0.957*	*0.754*
P23142	Fibulin-1	*CATPHGDN#ASLEATFVK*	*1.260*	*1.658*	*0.760*
Q08380	Galectin-3-binding protein	*AAIPSALDTN#SSK*	*0.499*	*0.667*	*0.747*
		*ALGFEN#ATQALGR*	*0.809*	*0.667*	*1.213*
		DAGVVCTN#ETR	0.659	0.667	0.987
		GLN#LTEDTYKPR	0.630	0.667	0.944
Q14624	Inter-alpha-trypsin inhibitor heavy chain H4	LPTQN#ITFQTESSVAEQ EAEFQSPK	0.865	0.737	1.173
Q99784	Noelin	*LDPVSLQTLQTWN#TSYPK*	*0.644*	*0.817*	*0.787*
		VQN#MSQSIEVLDR	0.759	0.817	0.928

a *The # denotes the residue site of N-glycosylation*.

b *Fold changes >1.5 or <0.667 were considered to be significant (highlighted in bold). Fold changes between 1.2 and 1.5 (1.2–1.5) or between 0.667 and 0.833 (0.667–0.833) were considered as minor (highlighted in italics)*.

EN#LTAPGSDSAVFFEQGTTR from ceruloplasmin and ALGFEN#ATQALGR from galectin-3-binding protein were reduced in N-glycosylated and parent protein levels, but they displayed increasing trends in N-glycosite occupancy. CATPHGDN#ASLEATFVK from fibulin-1 were up-regulated in N-glycosylated and parent protein levels, but N-glycosite occupancy of it was down-regulated. It was noteworthy that different N-glycosite from the same parent protein had the different occupancy, for example, AAIPSALDTN#SSK and ALGFEN#ATQALGR from galectin-3-binding protein, had fold changes of 0.747 and 1.213 in N-glycosite occupancy, respectively. In our previous study, N-glycosite occupancy of VVLHPN#YSQVDIGLIK was changed significantly in HCC patients compared with patients with hepatitis B virus infection (HBV) and liver cirrhosis (LC) (Zhang et al., 2012).

### Functional categories and patterns discovery

**Figure 5:**
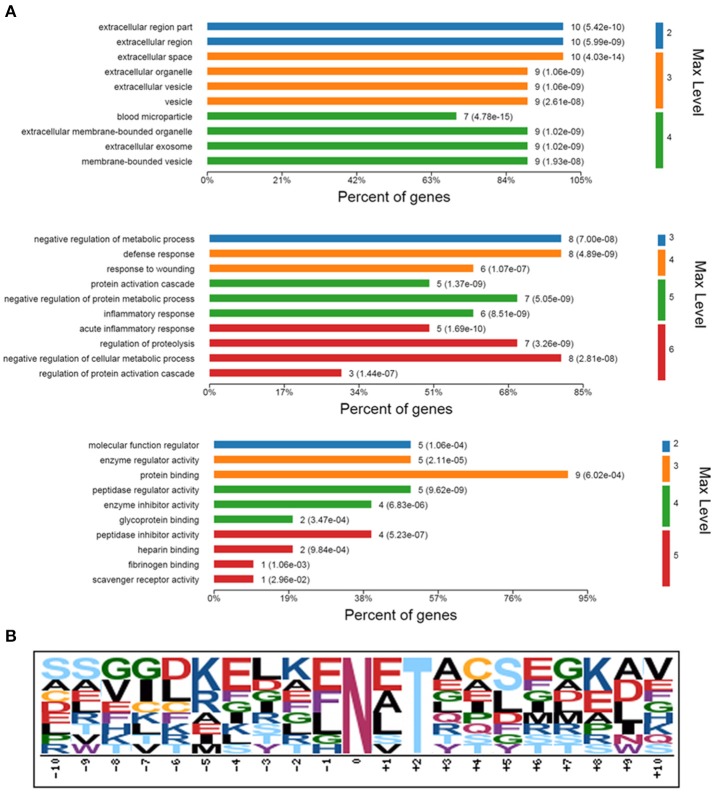
Bioinformatics analysis of identified 11 N-glycoproteins with changed N-glycosylation sites occupancy. **(A)** GO analysis for identified 11 N-glycoproteins. GO enriched were according to their cellular components, biological processes, and molecular functions. Max Level, maximal annotated level of this term in the GO graph (tree); P Value, calculated with Fish exact test with Hypergeometric algorithm; Count, number of genes/proteins in the query that are involved in this term. **(B)** A conserved motif of the 13 deglycosylated glycopeptides with different N-glycosylation sites occupancy was enriched by Motif-X.

In GO annotation, 11 serum N-glycoproteins with changed N-glycosite occupancy were categorized using OmicsBean according to their cellular components, biological processes, and molecular functions (Figure 5A). The annotation defined statistically significant with the P value which was calculated with Fish exact test with Hypergeometric algorithm. Most of the 11 N-glycoproteins were located in the blood microparticle or extracellular space, respectively. The related biological processes including acute inflammatory response (*p* = 1.69e-10), protein activation cascade (*p* = 1.37e-09), defense response (*p* = 4.89e-09), negative regulation of protein metabolic process (*p* = 5.05e-09), and so on. In molecular function annotation, the 11 N-glycoproteins were main associated with peptidase regulator activity (*p* = 9.62e-09), protein binding (*p* = 6.02e-04), glycoprotein binding (*p* = 3.47e-04) and scavenger receptor activity (*p* = 2.96e-02).

Motif-X was used to extract overrepresented motif of amino acids for the 13 deglycosylated glycopeptide with different N-glycosite occupancy from the 11 N-glycoproteins. Setting up “N” as central character, a conserved glycosylation motif was enriched by the created logo-like representations (Figure 5B).

**Figure 6:**
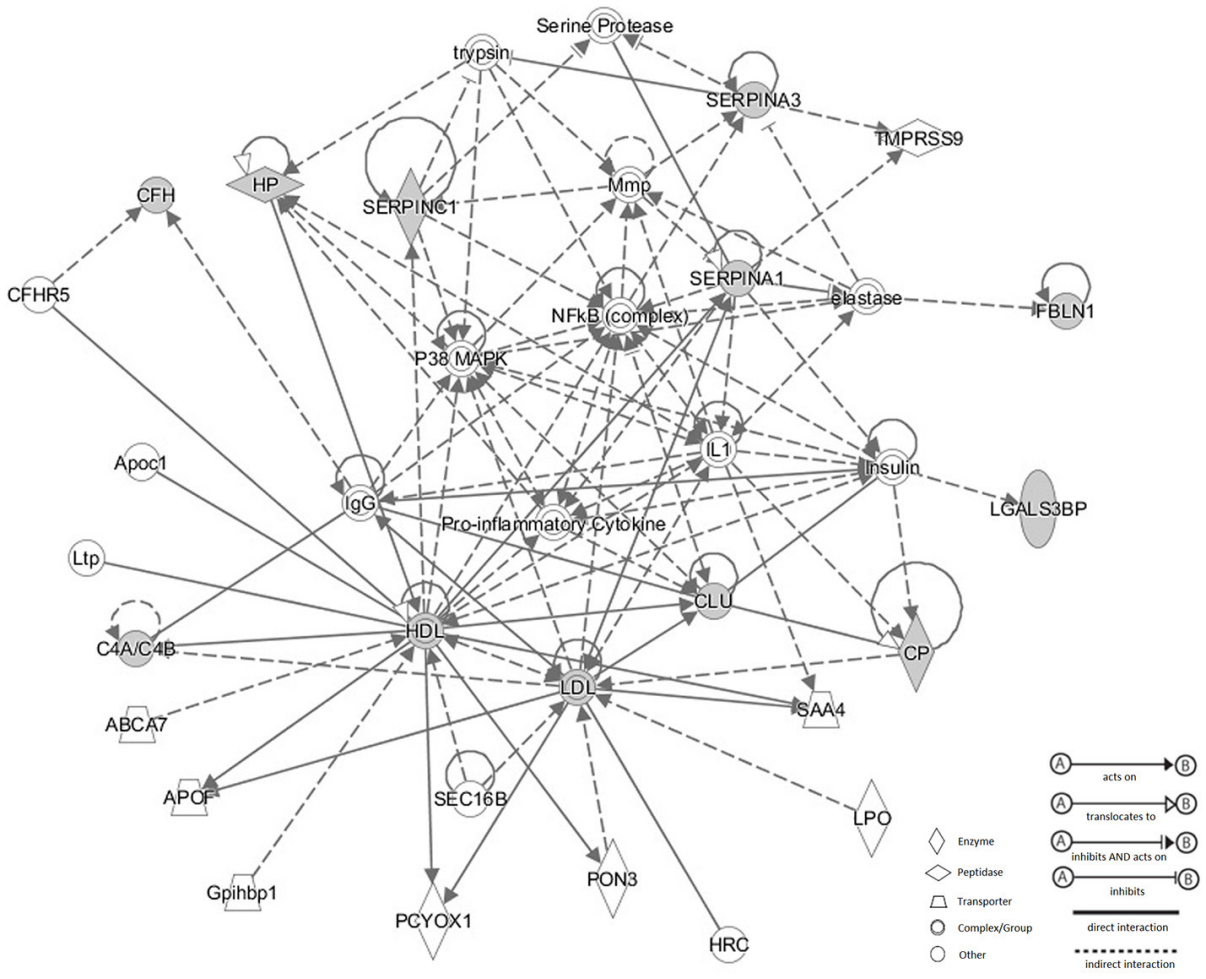
Corresponding network of these 11 N-glycoproteins were obtained using IPA analysis. There were four types of relationship in the network: acts on, translocates to, inhibits and acts on, inhibits. Full lines meant a direct interaction between two nodes, while the dotted lines meant an indirect interaction between two nodes. p38 MAPK and NF-κB were enriched in the network and these significantly changed N-glycoproteins were related to the two signaling pathways.

Corresponding network of these 11 N-glycoproteins were obtained using IPA analysis (Figure 6). Based on the Ingenuity Knowledge database, information about molecule-to-molecule interactions, biological networks and canonical pathways were collected and algorithmically generated. There were 5 different kinds of molecule shapes: enzyme, peptidase, transporter, complex/group and other. Relationships between two nodes were divided into 4 types, A: acts on, B: translocates to, C: inhibits and acts on and D: inhibits. Full lines in the network meant a direct interaction between two nodes, while the dotted lines meant an indirect interaction. According to the result, p38 MAPK and NF-κB were enriched in the network and these significantly changed N-glycoproteins were related to the two signaling pathways.

## Discussion

Glycoproteomics was critical to discover altered glycoproteins and glycans in occurrence and development of disease. Lectin microarray technology has been widely used for glycosylation studies in recent years (Kang et al., 2012; Li, Y. et al., 2013; Qin et al., 2013; Xin et al., 2014; Yang et al., 2015). It is a high-throughput technique which could reveal glycoforms using minimal sample preparation without release or derivatization of glycans and it could observe multiple and distinct binding interactions simultaneously (Pilobello et al., 2005; Fry et al., 2011). In this study, alterations of glycosylation between HCC patients with metastasis and those with non-metastasis were probed by this method. Poly-LacNAc or (GlcNAc)n binder LEL, exopolysaccharide binder NML, Sia binder PCL, β1,6-GlcNAc branched N-glycan binder PHA-L, GlcNAc binder STL, and (GlcNAc)n or multivalent Sia binder WGA showed significant increasing trend with strong binding in HCC patients with metastasis. Especially, PHA-L reactive structures were significantly changed. PHA-L could bind with β1,6-GlcNAc branched N-glycan which might play an important role in digestive cancers metastasis. Salomé et al. reported that the increasing β1,6 GlcNAc branched structures could decrease bisecting GlcNAc structures on E-cadherin molecule and lead to disruption of cell-cell contacts (Pinho et al., 2009). Qi et al. found that accumulation of this glycoform could result in enhanced cell migratory capacity by promoting PTPRT's dimerization and decreasing its catalytic activity (Qi et al., 2014).

Then, PHA-L affinity chromatography was applied to enrich the N-glycoproteins containing tetra-antennary complex-type N-glycan, followed by N-glycosite occupancy measurement with strategy of tandem ^18^O stable isotope labeling. This TOSIL strategy was reported to be advantageous for lowering the cost of experiment (Losfeld et al., 2012) and increasing mass shift (Nettleship et al., 2007). In our previous study, HGF was used to establish EMT model in Huh7 HCC cells. Lectin microarray analysis indicated that cell surface glycans of Huh7 were altered, for example, the binding abilities of PHA-L to glycan were elevated in EMT process (Li, S. et al., 2013). PHA-L could bind with β1,6-GlcNAc branched N-glycan, which is important in digestive cancers metastasis. β1,6-GlcNAc branched N-glycan was reported to be directly associated with metastasis (Dennis et al., 1987) and its specific increase could increase metastatic potential (Seberger and Chaney, 1999).

A total of 13 deglycosylated glycopeptides with changed N-glycosite occupancy were identified and 3 of them exhibited the different tendencies in N-glycosite occupancies compared to glycosylated and parent protein levels, including EN#LTAPGSDSAVFFEQGTTR of ceruloplasmin, CATPHGDN#ASLEATFVK of fibulin-1 and ALGFEN#ATQALGR of galectin-3-binding protein (Gal-3BP). CP was a copper-binding glycoprotein synthesized by the liver, had ferroxidase activity and would be an iron-regulatory protein. Occurrence, development and metastasis of HCC were consistently characterized by the lack of iron accumulation (Tan et al., 2009). CP could be considered as one of the potentially reliable biomarkers for the detection of HCC as its expression profiles was significantly differential and it also could be used in detecting liver metastasis from digestive cancer (Ferrin et al., 2015). Fibulin-1 was an extracellular matrix glycoprotein often associated with fibronectin and played an important role in cell adhesion and migration along protein fibers (Twal et al., 2001). It was validated that fibulin-1 was highly expressed on the surface of human gliomas and it might be involved in the aggressive nature of tumors (Towner et al., 2013). Gal-3BP is known as tumor-associated antigen 90 K or Mac-2 binding protein. It could combine with several galectins by glycan-dependent interactions and promote intergrin-mediated cell adhesion (Lin, T. W. et al., 2015). In this study, we found that N-glycosite occupancy of Gal-3BP was up-regulated and it might increase galectin-mediated tumor cell aggregation and then lead to increase the survival of cancer cells during the metastatic process. It is noteworthy that only albumin and IgG are deleted and detection of relatively low-abundant sera is affected and suppressed. In the future, we will try to delete most high-abundant proteins of sera and focus on low-abundant proteins.

Bioinformatics analysis was also performed for these 11 serum N-glycoproteins with changed N-glycosite occupancy. We speculated the related biological processes such as acute inflammatory response, protein activation cascade, defense response, negative regulation of protein metabolic process, which might be related with the metastasis of HCC. There were 2 significant nodes in the IPA network: p38 MAPK and NF-κB. Activation of p38 could promote metastasis and suppression of the p38 signaling pathway could inhibit cell migration and reduce the invasion of different tumor cells like gastric cancer cells, chondrosarcoma cells and colorectal cancer cells (Huang, Q. et al., 2014; Ren et al., 2014; Tsai et al., 2015; Yan et al., 2015). NF-κB, as a transcription factors, was frequently activated in tumors. According to previous reports, it could regulate cell migration, invasion and adhesion. Moreover, it was reported to be involved in tumor growth and progression (Tafani et al., 2013). A conserved motif of the 13 deglycosylated glycopeptides with altered N-glycosylation sites occupancy was enriched by Motif-X. This motif was showed as Asn-Xxx-Thr (NXT), and Xxx could be Glu (E), Ala (A), Leu (L), Ser (S), and Val (V). It indicated that occupancy changes of this conserved motif were more likely to occur in HCC with metastasis. It was reported that the occupancy changes of N-glycosylation site, such as NET and NVT of complement factor H, were associated with pancreatic ductal adenocarcinoma and chronic pancreatitis (Pan et al., 2014). Further study will need to uncover the frequency of these alterations and its mechanisms in diseases.

## Conclusion

In this study, PHA-L reactive structure (β1,6-GlcNAc branched N-glycan) was found to be increased significantly in HCC patients with metastasis compared with those with non-metastasis. Then, 11 PHA-L reactive glycoproteins with significantly changed N-glycosite occupancy were identified, which were associated with cell migration, invasion and adhesion through p38 MAPK and NF-κB signaling pathway. Alterations in N-glycosite occupancy were also related with HCC metastasis. β1,6 GlcNAc branching of N-glycans might be a metastasis-promoting glycoform and we believe quantification of changes in N-glycosite occupancy for PHA-L reactive glycoproteins in HCC metastasis serum could help to discover important glycoprotein of potential clinically significance as well as characterization of molecular mechanism of HCC metastasis.

## Author contributions

Study design: SZ, YL. Data acquisition and analysis: TL, SS, WL, XQ, LS. Interpretation of data: TL, SS, WL, LS, SZ, YL. Drafting and revising the work: TL, SS, SZ, YL. Final approval: TL, SS, WL, XQ, LS, SZ, YL. Agreement to be accountable of all aspects of the work: TL, SS, WL, XQ, LS, SZ, YL.

### Conflict of interest statement

The authors declare that the research was conducted in the absence of any commercial or financial relationships that could be construed as a potential conflict of interest.

## Supplementary material

Figure S1 Workflow for detecting glycoforms in serum glycoproteins by lectin microarray.

Figure S2 Workflow for quantifying N-glycosite occupancy in serum glycoproteins using TOSIL strategy.

Table S1 Name and binding specificity of 50 lectins used in lectin microarray.

## References

[B1] Alsenaidy M. A. Okbazghi S. Z. Kim J. H. Joshi S. B. Middaugh C. R. Tolbert T. J.. (2014). Physical stability comparisons of IgG1-Fc variants: effects of N-glycosylation site occupancy and Asp/Gln residues at site Asn 297. J. Pharm. Sci. 103, 1613–1627. 10.1002/jps.23975 24740840 PMC4512762

[B2] Apweiler R. Hermjakob H. Sharon N. (1999). On the frequency of protein glycosylation, as deduced from analysis of the SWISS-PROT database. Biochim. Biophys. Acta 1473, 4–8. 10.1016/S0304-4165(99)00165-8 10580125

[B3] Baboval T. Koul O. Smith F. I. (2000). N-glycosylation site occupancy of rat alpha-1,3-fucosyltransferase IV and the effect of glycosylation on enzymatic activity. Biochim. Biophys. Acta 1475, 383–389. 10.1016/S0304-4165(00)00094-5 10913840

[B4] Blomme B. Van Steenkiste C. Callewaert N. Van Vlierberghe H. (2009). Alteration of protein glycosylation in liver diseases. J. Hepatol. 50, 592–603. 10.1016/j.jhep.2008.12.010 19157620

[B5] Chou M. F. Schwartz D. (2011). Biological sequence motif discovery using motif-x. Curr. Protoc. Bioinformatics Chapter 13: Unit 13, 15–24. 10.1002/0471250953.bi1315s35 21901740

[B6] Comunale M. A. Wang M. Hafner J. Krakover J. Rodemich L. Kopenhaver B.. (2009). Identification and development of fucosylated glycoproteins as biomarkers of primary hepatocellular carcinoma. J. Proteome Res. 8, 595–602. 10.1021/pr800752c 19099421 PMC4427194

[B7] Dempsey E. Rudd P. M. (2012). Acute phase glycoproteins: bystanders or participants in carcinogenesis? Ann. N.Y. Acad. Sci. 1253, 122–132. 10.1111/j.1749-6632.2011.06420.x 22352780

[B8] Dennis J. W. Laferte S. Waghorne C. Breitman M. L. Kerbel R. S. (1987). Beta 1-6 branching of Asn-linked oligosaccharides is directly associated with metastasis. Science 236, 582–585. 10.1126/science.2953071 2953071

[B9] Ferrin G. Rodriguez-Peralvarez M. Aguilar-Melero P. Ranchal I. Llamoza C. Linares C. I.. (2015). Plasma protein biomarkers of hepatocellular carcinoma in HCV-infected alcoholic patients with cirrhosis. PLoS ONE 10:e0118527. 10.1371/journal.pone.0118527 25789864 PMC4366144

[B10] Fry S. A. Afrough B. Lomax-Browne H. J. Timms J. F. Velentzis L. S. Leathem A. J. (2011). Lectin microarray profiling of metastatic breast cancers. Glycobiology 21, 1060–1070. 10.1093/glycob/cwr045 21507904

[B11] Huang B. Sun L. Cao J. Zhang Y. Wu Q. Zhang J.. (2013). Downregulation of the GnT-V gene inhibits metastasis and invasion of BGC823 gastric cancer cells. Oncol. Rep. 29, 2392–2400. 10.3892/or.2013.2373 23563846

[B12] Huang B. Wu Q. Ge Y. Zhang J. Sun L. Zhang Y.. (2014). Expression of N-acetylglucosaminyltransferase V in gastric cancer correlates with metastasis and prognosis. Int. J. Oncol. 44, 849–857. 10.3892/ijo.2014.2248 24399258

[B13] Huang Q. Lan F. Wang X. Yu Y. Ouyang X. Zheng F.. (2014). IL-1beta-induced activation of p38 promotes metastasis in gastric adenocarcinoma via upregulation of AP-1/c-fos, MMP2 and MMP9. Mol. Cancer 13:18. 10.1186/1476-4598-13-18 24479681 PMC3937117

[B14] Jemal A. Bray F. Center M. M. Ferlay J. Ward E. Forman D. (2011). Global cancer statistics. CA Cancer J. Clin. 61, 69–90. 10.3322/caac.20107 21296855

[B15] Jia Y. L. Shi L. Zhou J. N. Fu C. J. Chen L. Yuan H. F.. (2011). Epimorphin promotes human hepatocellular carcinoma invasion and metastasis through activation of focal adhesion kinase/extracellular signal-regulated kinase/matrix metalloproteinase-9 axis. Hepatology 54, 1808–1818. 10.1002/hep.24562 22045676

[B16] Kang X. Wang N. Pei C. Sun L. Sun R. Chen J.. (2012). Glycan-related gene expression signatures in human metastatic hepatocellular carcinoma cells. Exp. Ther. Med. 3, 415–422. 10.3892/etm.2011.430 22969905 PMC3438600

[B17] Kannagi R. Izawa M. Koike T. Miyazaki K. Kimura N. (2004). Carbohydrate-mediated cell adhesion in cancer metastasis and angiogenesis. Cancer Sci. 95, 377–384. 10.1111/j.1349-7006.2004.tb03219.x 15132763 PMC11159147

[B18] Karve T. M. Cheema A. K. (2011). Small changes huge impact: the role of protein posttranslational modifications in cellular homeostasis and disease. J. Amino Acids 2011:207691. 10.4061/2011/207691 22312457 PMC3268018

[B19] Kim E. H. Misek D. E. (2011). Glycoproteomics-based identification of cancer biomarkers. Int. J. Proteomics 2011:601937. 10.1155/2011/601937 22084691 PMC3195811

[B20] Kim Y. S. Hwang S. Y. Kang H. Y. Sohn H. Oh S. Kim J. Y.. (2008). Functional proteomics study reveals that N-Acetylglucosaminyltransferase V reinforces the invasive/metastatic potential of colon cancer through aberrant glycosylation on tissue inhibitor of metalloproteinase-1. Mol. Cell. Proteomics 7, 1–14. 10.1074/mcp.M700084-MCP200 17878270

[B21] Kumada T. Toyoda H. Tada T. Kiriyama S. Tanikawa M. Hisanaga Y.. (2014). High-sensitivity Lens culinaris agglutinin-reactive alpha-fetoprotein assay predicts early detection of hepatocellular carcinoma. J. Gastroenterol. 49, 555–563. 10.1007/s00535-013-0883-1 24057163 PMC3953543

[B22] Kuzmanov U. Kosanam H. Diamandis E. P. (2013). The sweet and sour of serological glycoprotein tumor biomarker quantification. BMC Med. 11:31. 10.1186/1741-7015-11-31 23390961 PMC3751898

[B23] Li S. Mo C. Peng Q. Kang X. Sun C. Jiang K.. (2013). Cell surface glycan alterations in epithelial mesenchymal transition process of Huh7 hepatocellular carcinoma cell. PLoS ONE 8:e71273. 10.1371/journal.pone.0071273 23977005 PMC3748092

[B24] Li Y. Wen T. Zhu M. Li L. Wei J. Wu X.. (2013). Glycoproteomic analysis of tissues from patients with colon cancer using lectin microarrays and nanoLC-MS/MS. Mol. Biosyst. 9, 1877–1887. 10.1039/c3mb00013c 23567825

[B25] Lin T. W. Chang H. T. Chen C. H. Chen C. H. Lin S. W. Hsu T. L.. (2015). Galectin-3 binding protein and Galectin-1 interaction in breast cancer cell aggregation and metastasis. J. Am. Chem. Soc. 137, 9685–9693. 10.1021/jacs.5b04744 26168351

[B26] Liu Y. He J. Li C. Benitez R. Fu S. Marrero J.. (2010). Identification and confirmation of biomarkers using an integrated platform for quantitative analysis of glycoproteins and their glycosylations. J. Proteome Res. 9, 798–805. 10.1021/pr900715p 19961239 PMC2838716

[B27] Liu Z. Cao J. He Y. Qiao L. Xu C. Lu H.. (2010). Tandem 18O stable isotope labeling for quantification of N-glycoproteome. J. Proteome Res. 9, 227–236. 10.1021/pr900528j 19921957

[B28] Losfeld M. E. Soncin F. Ng B. G. Singec I. Freeze H. H. (2012). A sensitive green fluorescent protein biomarker of N-glycosylation site occupancy. FASEB J. 26, 4210–4217. 10.1096/fj.12-211656 22691915 PMC3448770

[B29] Mi W. Jia W. Zheng Z. Wang J. Cai Y. Ying W.. (2012). Surface glycoproteomic analysis of hepatocellular carcinoma cells by affinity enrichment and mass spectrometric identification. Glycoconj. J. 29, 411–424. 10.1007/s10719-012-9420-3 22752401

[B30] Nettleship J. E. Aplin R. Aricescu A. R. Evans E. J. Davis S. J. Crispin M.. (2007). Analysis of variable N-glycosylation site occupancy in glycoproteins by liquid chromatography electrospray ionization mass spectrometry. Anal. Biochem. 361, 149–151. 10.1016/j.ab.2006.11.005 17178093

[B31] Okuda K. Tanaka M. Kanazawa N. Nagashima J. Satomura S. Kinoshita H.. (1999). Evaluation of curability and prediction of prognosis after surgical treatment for hepatocellular carcinoma by lens culinaris agglutinin-reactive alpha-fetoprotein. Int. J. Oncol. 14, 265–271. 10.3892/ijo.14.2.265 9917501

[B32] Pan S. Chen R. Tamura Y. Crispin D. A. Lai L. A. May D. H.. (2014). Quantitative glycoproteomics analysis reveals changes in N-glycosylation level associated with pancreatic ductal adenocarcinoma. J. Proteome Res. 13, 1293–1306. 10.1021/pr4010184 24471499 PMC3993895

[B33] Pang R. W. Joh J. W. Johnson P. J. Monden M. Pawlik T. M. Poon R. T. (2008). Biology of hepatocellular carcinoma. Ann. Surg. Oncol. 15, 962–971. 10.1245/s10434-007-9730-z 18236113

[B34] Pilobello K. T. Krishnamoorthy L. Slawek D. Mahal L. K. (2005). Development of a lectin microarray for the rapid analysis of protein glycopatterns. Chembiochem 6, 985–989. 10.1002/cbic.200400403 15798991

[B35] Pinho S. S. Reis C. A. Paredes J. Magalhaes A. M. Ferreira A. C. Figueiredo J.. (2009). The role of N-acetylglucosaminyltransferase III and V in the post-transcriptional modifications of E-cadherin. Hum. Mol. Genet. 18, 2599–2608. 10.1093/hmg/ddp194 19403558

[B36] Qi J. Li N. Fan K. Yin P. Zhao C. Li Z.. (2014). beta1,6 GlcNAc branches-modified PTPRT attenuates its activity and promotes cell migration by STAT3 pathway. PLoS ONE 9:e98052. 10.1371/journal.pone.0098052 24846175 PMC4028250

[B37] Qin Y. Zhong Y. Zhu M. Dang L. Yu H. Chen Z.. (2013). Age- and sex-associated differences in the glycopatterns of human salivary glycoproteins and their roles against influenza a virus. J. Proteome Res. 12, 2742–2754. 10.1021/pr400096w 23590532

[B38] Rakus J. F. Mahal L. K. (2011). New technologies for glycomic analysis: toward a systematic understanding of the glycome. Annu. Rev. Anal. Chem. 4, 367–392. 10.1146/annurev-anchem-061010-113951 21456971

[B39] Ren H. Zhang S. Ma H. Wang Y. Liu D. Wang X.. (2014). Matrine reduces the proliferation and invasion of colorectal cancer cells via reducing the activity of p38 signaling pathway. Acta Biochim. Biophys. Sin. 46, 1049–1055. 10.1093/abbs/gmu101 25348737

[B40] Ren S. Zhang Z. Xu C. Guo L. Lu R. Sun Y.. (2016). Distribution of IgG galactosylation as a promising biomarker for cancer screening in multiple cancer types. Cell Res. 26, 963–966. 10.1038/cr.2016.83 27364686 PMC4973333

[B41] Sato Y. Nakata K. Kato Y. Shima M. Ishii N. Koji T.. (1993). Early recognition of hepatocellular carcinoma based on altered profiles of alpha-fetoprotein. N. Engl. J. Med. 328, 1802–1806. 10.1056/NEJM199306243282502 7684823

[B42] Schwartz D. Gygi S. P. (2005). An iterative statistical approach to the identification of protein phosphorylation motifs from large-scale data sets. Nat. Biotechnol. 23, 1391–1398. 10.1038/nbt1146 16273072

[B43] Seberger P. J. Chaney W. G. (1999). Control of metastasis by Asn-linked, beta1-6 branched oligosaccharides in mouse mammary cancer cells. Glycobiology 9, 235–241. 10.1093/glycob/9.3.235 10024661

[B44] Seelentag W. K. Li W. P. Schmitz S. F. Metzger U. Aeberhard P. Heitz P. U.. (1998). Prognostic value of beta1,6-branched oligosaccharides in human colorectal carcinoma. Cancer Res. 58, 5559–5564. 9850094

[B45] Shah A. K. Cao K. A. Choi E. Chen D. Gautier B. Nancarrow D.. (2015). Serum glycoprotein biomarker discovery and qualification pipeline reveals novel diagnostic biomarker candidates for esophageal adenocarcinoma. Mol. Cell. Proteomics 14, 3023–3039. 10.1074/mcp.M115.050922 26404905 PMC4638044

[B46] Stavenhagen K. Plomp R. Wuhrer M. (2015). Site-specific protein N- and O-glycosylation analysis by a C18-porous graphitized carbon-liquid chromatography-electrospray ionization mass spectrometry approach using pronase treated glycopeptides. Anal. Chem. 87, 11691–11699. 10.1021/acs.analchem.5b02366 26536155

[B47] Sumer-Bayraktar Z. Nguyen-Khuong T. Jayo R. Chen D. D. Ali S. Packer N. H.. (2012). Micro- and macroheterogeneity of N-glycosylation yields size and charge isoforms of human sex hormone binding globulin circulating in serum. Proteomics 12, 3315–3327. 10.1002/pmic.201200354 23001782

[B48] Tafani M. Pucci B. Russo A. Schito L. Pellegrini L. Perrone G. A.. (2013). Modulators of HIF1α and NFkB in cancer treatment: is it a rational approach for controlling malignant progression? Front. Pharmacol. 4:13. 10.3389/fphar.2013.00013 23408731 PMC3569619

[B49] Tan M. G. Kumarasinghe M. P. Wang S. M. Ooi L. L. Aw S. E. Hui K. M. (2009). Modulation of iron-regulatory genes in human hepatocellular carcinoma and its physiological consequences. Exp. Biol. Med. 234, 693–702. 10.3181/0807-RM-227 19307463

[B50] Towner R. A. Jensen R. L. Vaillant B. Colman H. Saunders D. Giles C. B.. (2013). Experimental validation of 5 *in-silico* predicted glioma biomarkers. Neuro-oncology 15, 1625–1634. 10.1093/neuonc/not124 24158112 PMC3829592

[B51] Tsai C. H. Tsai H. C. Huang H. N. Hung C. H. Hsu C. J. Fong Y. C.. (2015). Resistin promotes tumor metastasis by down-regulation of miR-519d through the AMPK/p38 signaling pathway in human chondrosarcoma cells. Oncotarget 6, 258–270. 10.18632/oncotarget.2724 25404641 PMC4381593

[B52] Tung-Ping Poon R. Fan S. T. Wong J. (2000). Risk factors, prevention, and management of postoperative recurrence after resection of hepatocellular carcinoma. Ann. Surg. 232, 10–24. 10.1097/00000658-200007000-00003 10862190 PMC1421103

[B53] Twal W. O. Czirok A. Hegedus B. Knaak C. Chintalapudi M. R. Okagawa H.. (2001). Fibulin-1 suppression of fibronectin-regulated cell adhesion and motility. J. Cell Sci. 114(Pt 24), 4587–4598. 11792823 10.1242/jcs.114.24.4587

[B54] Varki A. Lowe J. B. (2009). Biological roles of glycans, in Essentials of Glycobiology, eds Varki A. Cummings R. D. Esko J. D. Freeze H. H. Stanley P. Bertozzi C. R. Hart G. W. Etzler M. E. (Cold Spring Harborm NY: The Consortium of Glycobiology Editors, La Jolla, California).

[B55] Wang K. Zhao Y. Li M. Gao F. Yang M. K. Wang X.. (2014). Analysis of phosphoproteome in rice pistil. Proteomics 14, 2319–2334. 10.1002/pmic.201400004 25074045

[B56] Xia Y. Yan Z. L. Xi T. Wang K. Li J. Shi L. H.. (2012). A case-control study of correlation between preoperative serum AFP and recurrence of hepatocellular carcinoma after curative hepatectomy. Hepatogastroenterology 59, 2248–2254. 10.5754/hge11978 22366528

[B57] Xin A. J. Cheng L. Diao H. Wang P. Gu Y. H. Wu B.. (2014). Comprehensive profiling of accessible surface glycans of mammalian sperm using a lectin microarray. Clin. Proteomics 11:10. 10.1186/1559-0275-11-10 24629138 PMC4003823

[B58] Xu Y. Bailey U. M. Schulz B. L. (2015). Automated measurement of site-specific N-glycosylation occupancy with SWATH-MS. Proteomics 15, 2177–2186. 10.1002/pmic.201400465 25737293

[B59] Yamamoto J. Okada S. Shimada K. Okusaka T. Yamasaki S. Ueno H.. (2001). Treatment strategy for small hepatocellular carcinoma: comparison of long-term results after percutaneous ethanol injection therapy and surgical resection. Hepatology 34(4 Pt 1), 707–713. 10.1053/jhep.2001.27950 11584366

[B60] Yan X. Rui X. Zhang K. (2015). Baicalein inhibits the invasion of gastric cancer cells by suppressing the activity of the p38 signaling pathway. Oncol. Rep. 33, 737–743. 10.3892/or.2014.3669 25502212

[B61] Yang G. Tan Z. Lu W. Guo J. Yu H. Yu J.. (2015). Quantitative glycome analysis of N-glycan patterns in bladder cancer vs normal bladder cells using an integrated strategy. J. Proteome Res. 14, 639–653. 10.1021/pr5006026 25536294

[B62] Yang H. Fang F. Chang R. Yang L. (2013). MicroRNA-140-5p suppresses tumor growth and metastasis by targeting transforming growth factor beta receptor 1 and fibroblast growth factor 9 in hepatocellular carcinoma. Hepatology 58, 205–217. 10.1002/hep.26315 23401231

[B63] Zhang M. Chen G. X. Lv D. W. Li X. H. Yan Y. M. (2015). N-linked glycoproteome profiling of seedling leaf in Brachypodium distachyon L. J. Proteome Res. 14, 1727–1738. 10.1021/pr501080r 25652041

[B64] Zhang S. Liu X. Kang X. Sun C. Lu H. Yang P.. (2012). iTRAQ plus 18O: a new technique for target glycoprotein analysis. Talanta 91, 122–127. 10.1016/j.talanta.2012.01.033 22365690

[B65] Zhang S. Shang S. Li W. Qin X. Liu Y. (2016). Insights on N-glycosylation of human haptoglobin and its association with cancers. Glycobiology 26, 684–692. 10.1093/glycob/cww016 26873173

