# Supplementary figures and images for: Assessment of Hepatocellular Carcinoma Metastasis Glycobiomarkers Using Advanced Quantitative N-glycoproteome Analysis
Source: Front Physiol. 2017 Jul 7;8:472. doi: 10.3389/fphys.2017.00472 (PMC5500640; doi:10.3389/fphys.2017.00472)

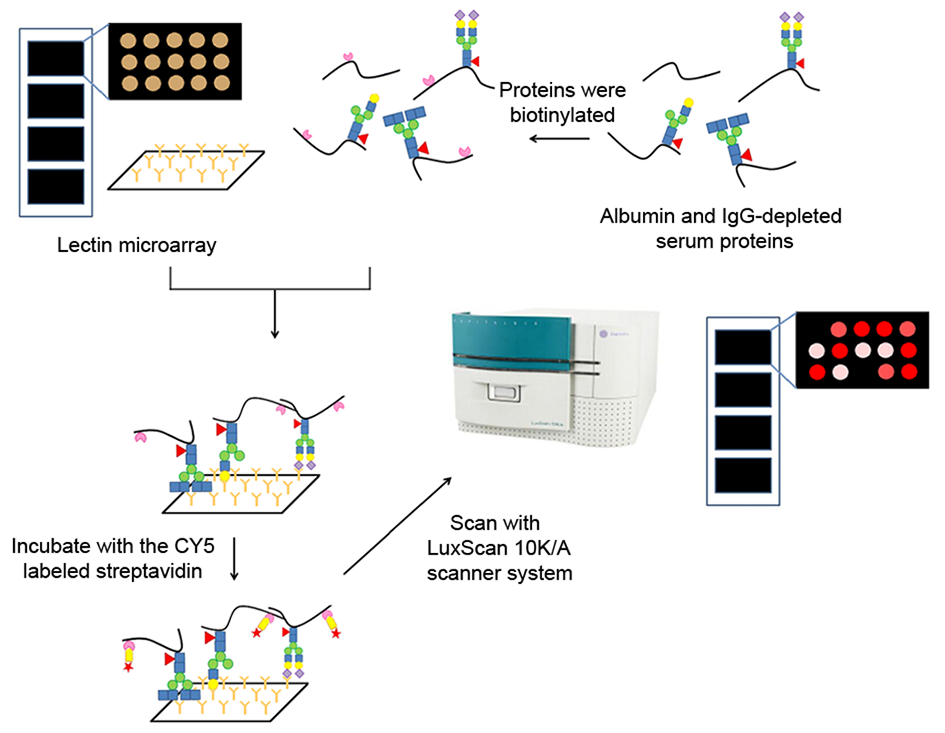

Supplement: Figure S1 — Workflow for detecting glycoforms in serum glycoproteins by lectin microarray. [file Image1.TIF]

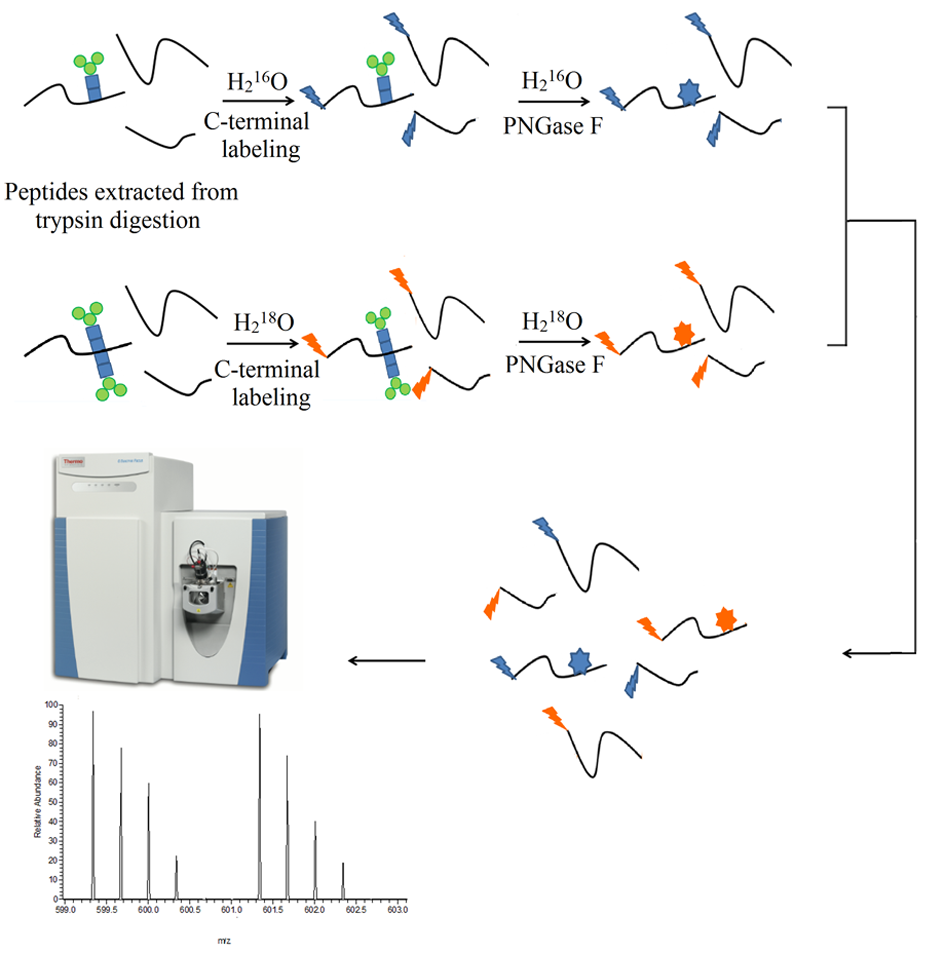

Supplement: Figure S2 — Workflow for quantifying N-glycosite occupancy in serum glycoproteins using TOSIL strategy. [file Image2.TIF]
