# Supplementary material for: Assessment of Hepatocellular Carcinoma Metastasis Glycobiomarkers Using Advanced Quantitative N-glycoproteome Analysis
Source: Front Physiol. 2017 Jul 7;8:472. doi: 10.3389/fphys.2017.00472 (PMC5500640; doi:10.3389/fphys.2017.00472)
Supplement: Table S1 — Name and binding specificity of 50 lectins used in lectin microarray. [file Table1.DOCX]

**Table S1 Name and binding specificity of 50 lectins used in the lectin microarray**

| **Carbohydrate** | **Name** | **Abbreviation** | **Specific binding carbohydrates** |
| --- | --- | --- | --- |
| Fuc | Aleuria aurantia lectinn | AAL | Terminal Fucα1-6GlcNAc,Fucα1-3Galβ1-4GalNAc |
|  | Lens culinaris agglutinin | LCA | Fucα1-6GlcNAc(core) |
|  | Lotus tetragonolobus lectin | LTL | Fucα1-3GlcNAc(core), sLe^x^, Le^x^ |
|  | Pisum sativum agglutinin | PSA | Fucα-N-acetylchitobiose-Man |
|  | Ulex europaeus agglutinin | UEA | Fucα1-2Galβ1-4GlcNAc |
| Galβ1-3GalNAc | Agaricus bisporus lectin | ABL | Galβ1-3GalNAcα-Ser/Thr(T), Siaα2-3(6)Galβ1-3GalNAcα-Ser/Thr |
|  | Amaranthus caudatus lectin | ACL | Galβ1-3GalNAcα-Ser/Thr(T) |
|  | Bauhinia purpurea lectin | BPL | Galβ1-3GalNAc |
|  | Jacalin | JAC | Galβ1-3GalNAcα-Ser/Thr(T), GalNAcα-Ser/Thr(T) |
|  | Peanut agglutinin | PA | Galβ1-3GalNAc |
| GalNAc/Gal | Caragana arborescens lectin | CAL | GalNAc |
|  | Codium fragile lectin | CFL | GalNAc |
|  | Cytisus scoparius Lectin | CSL | Gal, GalNAc |
|  | Dolichos biflorus agglutinin | DBA | GalNAcα-Ser/Thr(Tn), GalNAcβ1-3Gal |
|  | Euonymus europaeus lectin | EEL | Galα1-3(Fucα1-2)Gal |
|  | Griffonia simplicifolia lectin I | GSL I | α-GalNAc,α-Gal, GalNAcα-Ser/Thr(Tn) |
|  | GSL I - isolectin B4 | GSL1b4 | α-GalNAc, α-Gal |
|  | Griffonia simplicifolia lectin II | GSL II | GalNAc, Gal |
|  | Helix aspersa lectin | HAL | GalNAc |
|  | Helix pomatia lectin | HPL | α-GalNAc |
|  | Maclura pomifera lectin | MPL | αGalNAc |
|  | Psophocarpus tetragonolobus lectin I | PTL I | α-GalNAc, Gal |
|  | Psophocarpus tetragonolobus lectin II | PTL II | Gal |
|  | Ricinus communis agglutinin-I | RCA-I | Gal, GalNAc |
|  | Ricinus communis agglutinin60 | RCA 60 | Gal, GalNAc |
|  | Ricin B Chain | RIC | Gal, GalNAc |
|  | Soybean agglutinin | SBA | Terminal GalNAc (especially GalNAcα1-3Gal) |
|  | Sophora japonica agglutinin | SJA | Gal, Terminal GalNAc |
|  | Viscum album lectin | VAL | β-Gal |
|  | Vicia villosa lectin | VVL | GalNAc, GalNAcα-Ser/Thr(Tn) |
|  | Wisteria floribunda lectin | WFL | GalNAcα/β1-3/6Gal |
| Man | Concanavalin A | Con A | Branched and terminal Man, terminal GlcNAc |
|  | Galanthus nivalis lectin | GNL | Terminal α1-3 Man |
|  | Hippeastrum hybrid lectin | HHL | Non-substituted α-1,6Man |
|  | Naja mossambica lectin | NML | Man |
|  | Narcissus pseudonarcissus lectin | NPL | Non-substituted α-1,6Man |
|  |  |  | GalNAcα-Ser/Thr(Tn), |
| GlcNAc | Datura stramonium agglutinin | DSA | GlcNAc |
|  | Lycopersicon esculentum lectin | LEL | Poly-LacNAc, (GlcNAc)_n_ |
|  | Phytolacca americana  lectin | PAL | β-GlcNac |
|  | Phytolacca americana lectin | PWA | GlcNAcβ1-4GlcNAc, Galβ1-4GlcNAc |
|  | Solanum tuberosum lectin | STL | (GlcNAc)_n_ |
|  | Wheat germ agglutinin | WGA | (GlcNAc)_n_, Multivalent Sia |
| Galβ1-4GlcNAc | Erythrina cristagalli lectin | ECL | Galβ1-4GlcNAc |
| Sia | Maackia amurensis lectin-I | MAL-I | Siaα2-3Gal, Galβ1-4GlcNAc |
|  | Maackia amurensis lectin-II | MAL-II | Siaα2-3Gal, Galβ1-4GlcNAc |
|  | Phaseolus coccineus lectin | PCL | Sialic acid, (GlcNAc)_n_ |
|  | Sambucus nigra lectin | SNA | Siaα2-6Galβ1-4GlcNAc |
| Complex | Phaseolus vulgaris Erythroagglutinin | PHA-E | Bisecting GlcNAc and Biantennary N-glycans |
|  | Phaseolus vulgaris Leucoagglutinin | PHA-L | β1,6-GlcNAc branched |
| Other | Limulus polyphemus lectin | LPL | N-acetylated D-hexosamines |
